# A deep learning model to detect novel pore-forming proteins

**DOI:** 10.1038/s41598-022-05970-w

**Published:** 2022-02-07

**Authors:** Theju Jacob, Theodore W. Kahn

**Affiliations:** BASF Corporation, 3500 Paramount Parkway, Morrisville, NC 27560 USA

**Keywords:** Computational biology and bioinformatics, Protein analysis, Protein function predictions

## Abstract

Many pore-forming proteins originating from pathogenic bacteria are toxic against agricultural pests. They are the key ingredients in several pesticidal products for agricultural use, including transgenic crops. There is an urgent need to identify novel pore-forming proteins to combat development of resistance in pests to existing products, and to develop products that are effective against a broader range of pests. Existing computational methodologies to search for these proteins rely on sequence homology-based approaches. These approaches are based on similarities between protein sequences, and thus are limited in their usefulness for discovering novel proteins. In this paper, we outline a novel deep learning model trained on pore-forming proteins from the public domain. We compare different ways of encoding protein information during training, and contrast it with traditional approaches. We show that our model is capable of identifying known pore formers with no sequence similarity to the proteins used to train the model, and therefore holds promise for identifying novel pore formers.

## Introduction

Pore-forming proteins form conduits in cell plasma membranes, allowing intracellular and extracellular solutes to leak across cell boundaries. Although the amino acid sequences and three-dimensional structures of pore-forming proteins are extremely diverse, they share a common mode of action in which water-soluble monomers come together to form oligomeric pre-pore structures that insert into membranes to form pores^1^. Many pore formers originating from pathogenic bacteria are well documented to be toxic against agricultural pests^2,3^. They operate by forming pores in the gut cell membranes of the pests once ingested, causing the death of the pests.

Orally active pore formers are the key ingredients in several pesticidal products for agricultural use, including transgenic crops^4^. A wide variety of pore-forming protein families are needed for this application, for two reasons. First, any given pore former is typically only active against a small number of pest species^5^. As a result, proteins from more than one family may be needed to protect a crop from its common pests. Second, the wide-spread use of a particular protein can lead to the development of pests that are resistant to that protein—for instance, because of a modification to the insect receptor to which the toxic protein binds^6–8^. There is hence an urgent need to identify novel pore formers that can then be developed into new products that will control a broader range of pests, and will delay the development of resistance in pests. A pore former with a new site of action/receptor would overcome resistance, and combining multiple sites or modes of action in one product can delay the development of resistance. Novel pore formers are difficult to find by traditional methods, which involve feeding bacterial cultures to pests, or searching for homologs of known pore formers^9^. Modern genome sequencing methods have generated a vast untapped resource of genes whose function is unknown^10–12^. Since testing more than a tiny fraction of them for pore-forming activity experimentally is not feasible, computational methods are needed to prioritize which of these proteins should be tested.

The current computational methodology for detecting novel pore-forming proteins relies on sequence homology-based approaches. Sequences of entire proteins and of protein domains from known pore-forming proteins are compared with those proteins whose functionality is unknown, and those that are similar to known toxins are shortlisted for further testing. BLAST^13^ and Hidden Markov Models^14^ are the most widely employed tools for sequence homology comparisons. However, these methods (1) capture only dependencies between amino acids that are within short distances along the protein sequence, and (2) capture only sequences that are fairly similar to already existing pore formers. Truly novel pore formers may be sufficiently different from known pore formers that these methods would not identify them.

**Figure 1:**
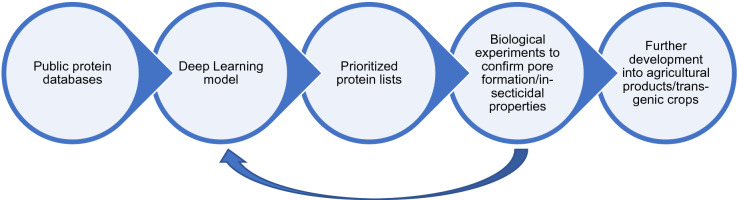
The usage of the proposed deep learning model in a workflow that could give rise to the next generation of agricultural products. In the proposed workflow, the deep learning model serves to shortlist proteins for further testing, thereby saving time and resources that need to be committed for experiments in laboratory and field settings. We can use our learnings from the experiments to further refine the model.

We seek to build a model that will enable us to move beyond sequence homology in detecting potential new pore-forming toxins, in the absence of 3-dimensional structural data for either the known or the potentially novel toxins. Deep learning models are increasingly used for a variety of tasks related to proteins^15–19^. By building a deep learning model, we seek to capture not just dependencies between neighboring amino acids as is done in traditional sequence matching methods like HMMs, but also dependencies between amino acids that are farther apart along the protein sequence. By encoding amino acids in terms of their physical and chemical properties, we also hope to capture the basic characteristics of a protein that form pores, allowing us to identify novel pore formers based on similarities that currently are not recognized. The expectation is that our model will serve as a tool to prioritize proteins of previously unknown function for further testing in lab experiments for their pore forming and insecticidal activity. The throughput limitations of lab experiments make it impractical to test all proteins of unknown function unless there is a way to prioritize those most likely to have the desired activity. Any of the proteins prioritized by our model that were confirmed to have activity in biological experiments could then be developed into potential agricultural products. This process is outlined in Fig. 1.

Pore forming proteins can be broadly classified into alpha and beta categories based on the secondary structures of their membrane spanning elements^20,21^. Examples of pesticidal alpha pore formers include multiple Cry protein family members and Vip3 protein family members, while examples of pesticidal beta pore formers include Mtx and Toxin 10 protein family members^20,22^. In this paper, we ask the question: in the absence of widely available structure data, what can we learn from comparing the sequences of pore-forming proteins across the board, from both categories combined? Can we build a machine learning model that can learn to distinguish pore-forming proteins from non-pore formers, regardless of what category they may belong to? Towards this end, we downloaded sequences of alpha and beta pore-forming proteins from Uniprot^23^ and used them as our training set for a deep learning model. We used a series of encoding methods for the proteins in our training set, and evaluated their accuracy in distinguishing pore forming from non-pore forming proteins. We also evaluated the precision and recall characteristics of these encoding methods. In addition, we compared our methods to BLAST and HMM models when attempting to detect pore formers that were not part of the training set.

In the subsequent sections, we discuss the details of our model, and list the data sources that we used to train our model. This is followed by a discussion on the various encoding schemes we evaluated. The results section outlines the accuracy and loss curves obtained during model training, and also the receiver operating characteristic curve of the best variation of our model. We then compare our model with BLAST and HMM, in terms of the number of novel pore formers it is able to pick up from a set of proteins not seen during training. We also show that our model correctly classifies as negative proteins those that have been annotated as non-pore forming. We conclude by stating why our approach can be a valuable aid in the development of a new generation of agricultural products.

## Methods

### Model

**Figure 2:**
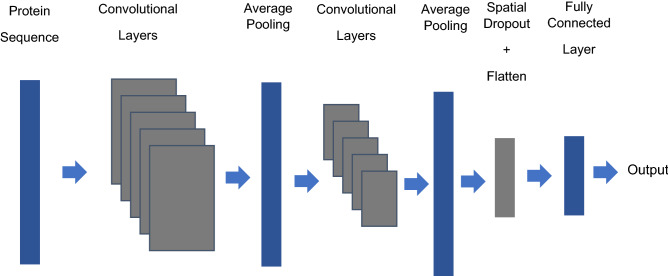
Outline of the model: the encoded protein sequence is fed to convolutional layers with 25 filters of dimensions 1 × 100. The second set of convolutional layer filters have dimensions 1 × 50. ReLU was used as the activation function. Mean squared error was the metric used as the loss function. The pooling layers had a pool size of 5, and the dropout layer had a factor of 0.25.

The outline of the deep learning model is as shown in Fig. 2. The encoded protein sequence passes through multiple convolutional and pooling layers. It is then followed by a dropout layer, after which it is passed through a fully connected layer to the output. The output indicates the probability that a given protein is a pore former, with a range from 0 to 1. The hyperparameters of the network were selected by Bayesian optimization on the training data set.

The model was developed in Python 3.7, using Keras and Scikit package^24–26^. They were run on a Linux computational cluster. The training and evaluation of the model could be completed in less than 24 h.

### Data

We used Uniprot^23^ as our source for alpha and beta pore-forming proteins. Proteins that are currently used for insect pest control in transgenic plants commercially are pore formers, and they are relatively large proteins that can be expressed in plants and broken down in vertebrate digestive systems. They interact with receptors in the target pests before forming pores, giving them relatively narrow specificity^3,27^. Because of these many requirements, not very many of them are available for us to train a computational model that can shortlist proteins that are potentially pore formers and insecticidal. To increase the size of the training set we therefore included pore formers that are not necessarily insecticidal in our data set. Our workflow seeks to use the computational model we build to search for truly novel proteins whose functions are currently unknown, that are predicted to likely be pore formers. Then we will experimentally test them for pore forming activity, followed by testing for insecticidal properties. Such a workflow will increase the chances of finding truly novel insecticidal proteins, but we expect only a small subset of the predicted pore-forming proteins selected by the model to be insecticidal. Starting with the search for pore-forming activity would ensure that we are considering a broad enough range of potential candidate proteins, while giving us a decent sized data set to train our model.

Under alpha pore formers, we included pesticidal crystal proteins, actinoporins, hemolysins, and colicins. Under beta pore formers, we included leucocidins, alpha-hemolysins, perifringolysins, aerolysins, haemolysins, and cytolysins. We eliminated all sequences that were shorter than 50 or longer than 2000 amino acids. These restrictions were put in place as the proteins that are typically used in transgenic plants fall within that size range^4^. Antibiotic peptides and other pore- forming peptides were excluded by setting a lower limit on the size of the proteins that we considered.

We included both fragments and full proteins in our data set. Fragments that were included contained protein domains that were known to be found in insecticidal proteins, hence were deemed significant. We obtained approximately 3000 proteins belonging to both alpha and beta pore-forming families. We clustered the sequences at 70% identity before training, to avoid overfitting of our model. Post-clustering, we were left with approximately 2000 proteins in the data set. We used zero padding to ensure all sequences were of the same length before training. This step also enabled us to avoid multiple sequence alignments that would have rendered our model impractical when eventually testing with millions of proteins.

For our negative training set, we wanted to cover as much diversity as possible in terms of possible protein structures the model might encounter. We used a culled PDB dataset from the PISCES server^28^. The dataset sequences had less than 20 percent sequence identity, with better than 1.8 Å resolution. The lengths were once again restricted to fall within the 50–2000 amino acid range. We eliminated sequences that were similar to the ones in our positive training set, based on BLASTP results with an E-value of 0.01. The final list had approximately 5000 sequences. Elimination by sequence-based approaches does not guarantee that there are no pore formers in the negative set, but all of the retained proteins have been annotated as having functionality other than pore formation. We expect that novel pore formers predicted by the model will be proteins that have no existing annotations, like hypothetical proteins.

### Comparison of various encoding schemes

Protein sequences consist of amino acids, typically denoted by letters. For a computational algorithm to make sense of them, they need to be represented as numbers. A representation of letters along the protein sequence by predetermined numbers will work—for example, every amino acid can be represented by a unique number. Or they can be one-hot encoded, where every position along a protein sequence is represented by an indicator array, with a one denoting the amino acid in that position, and the rest all zeros. In the literature, a method commonly used is the representation of a combination of, say, amino acids in sets of three (trigrams), by a unique number^15^. Position specific scoring matrices (PSSM) is another widely used method to obtain numerical representations for protein sequences^29^.

In this work, we sought to represent protein sequences by an encoding method that would enable us to eventually test our model with millions of test proteins. This ruled out methods that required comparisons with existing protein databases, like PSSMs. We also ruled out utilizing domain information from known pore formers, to avoid biasing our model towards already known proteins. One-hot encoding would enable us to rapidly convert the amino acid sequences to numbers, but it treats all amino acids the same. We therefore sought a method of representing amino acids that captures their properties in as low dimensional a space as possible. After evaluating multiple methods outlined in the literature, we eventually chose the representation outlined in^30^. In the work by Atchley et al., 54 selected amino acid attributes were analyzed and reduced to 5 factors. The 5 numbers that corresponded to each amino acid captured:

1. Accessibility, polarity, hydrophobicity
2. Propensity for secondary structure
3. Molecular size
4. Codon composition
5. Electrostatic charge

Similar numbers along any of these 5 factors indicated similarity in the corresponding property space. In addition to capturing amino acid properties, the Atchley et al. representation is attractive for our purposes as the feature space is comparatively low dimensional. For example, one-hot encoding represents an amino acid using a 28-dimensional array (all of the amino acids plus characters used for zero padding), while the Atchley method encodes the same amino acid using only a 5-dimensional array. A smaller feature space makes the training times and memory requirements of the model much more manageable, but we attempted to strike a balance with accuracy and loss metrics as well. In this paper, we evaluated one-hot encoding (28 dimensional feature space), Atchley encoding (5-dimensional feature space), as well as combined one-hot encoding and Atchley encoding (33 dimensional feature space) methods. For Atchley encoding, we represented every amino acid by the 5 numbers that were found in the previous publication by Atchley et al. We did not do the reduction of the amino acid attributes to 5 numbers ourselves.

## Results

**Figure 3:**
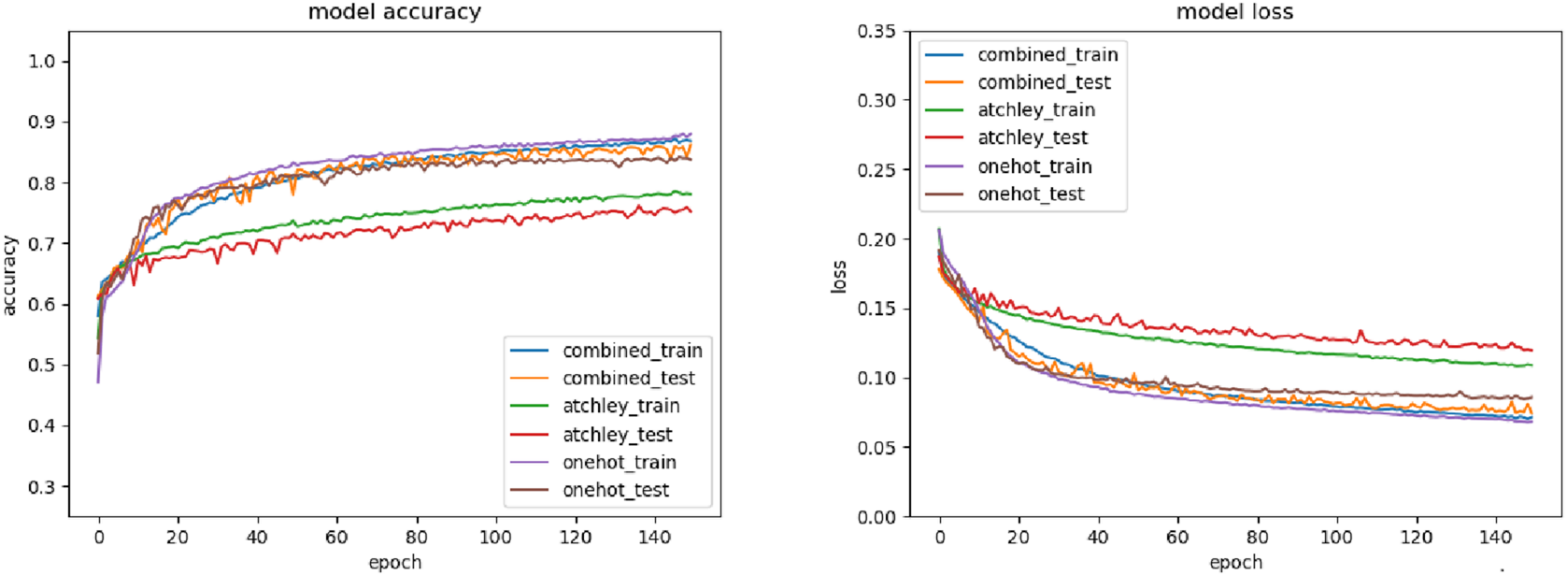
Accuracy and loss curves for one-hot encoding, Atchley 5-factor encoding, and combined encoding methods.

We evaluated three different encoding schemes—Atchley, one hot, and their combination. For definition of these encoding schemes, see the methods section. Accuracy and loss curves for the different encoding methods are shown in Fig. 3. As can be observed, the accuracy and loss curves converged during training of the model. Accuracy values reaching approximately 90% and loss values reaching approximately 5% were observed by end of training. One-hot and the combined encoding methods did better than Atchley encoding in terms of both accuracy as well as loss curves. The combined encoding method was comparable to one-hot encoding initially, but towards the end of the training, started to give better performance than one-hot encoding. We split our data set into 80:20 for training and testing purposes respectively. The split was randomized, and multiple trials were conducted to ensure that the accuracy and loss values were consistent.

**Figure 4:**
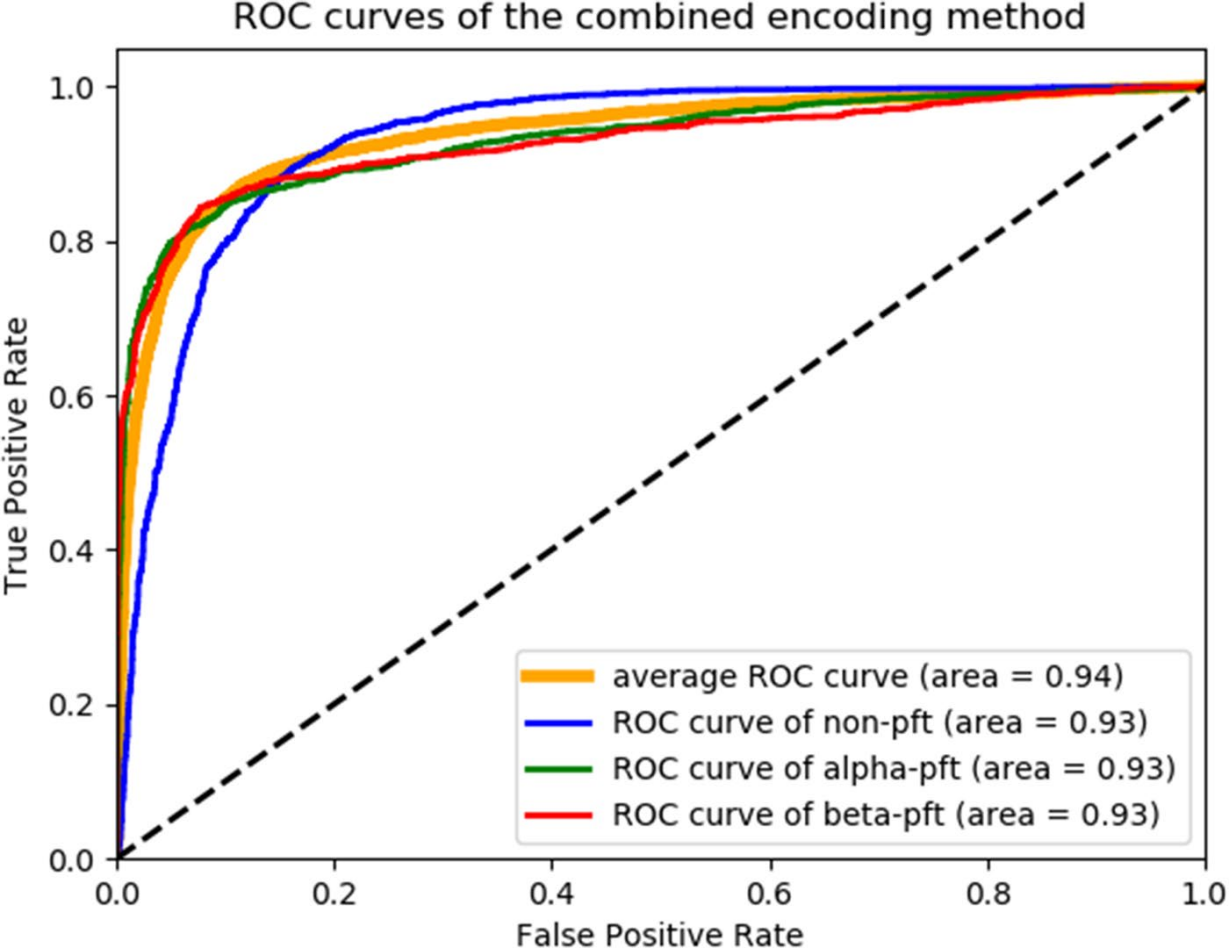
Receiver operating characteristic curves of the combined encoding method. We show the curves for the negative, alpha, and beta pore formers, as well as the average ROC curve.

The ROC curves for combined one-hot-Atchley encoding method are shown in Fig. 4. As can be seen from the curves and the area under the curve (auc) values, the model gives near ideal performance on the dataset it was trained with.

Our model is proposed as a first step for short listing proteins that are potentially pore forming. Their pore forming and insecticidal abilities would need to be ascertained further in lab settings. Since lab experiments tend to be time and resource consuming, it is not feasible to screen every hypothetical protein from protein databases—a problem our model is intended to ease. As a test of whether our model will be useful for finding proteins that have not previously been recognized to be pore formers, we evaluated whether it is capable of identifying known pore formers it had not seen during training, and whether it is better at that task than standard methods like BLAST and HMM.. To that end, we considered 3 known pore former families that we had not considered during training of the model—Vip3, MACPF, and Toxin 10. A comparison of the performance of the model against BLAST and HMM is summarized in Table 1.

**Table 1:** Table comparing BLAST, HMM, and our model with the three protein families of interest.

Protein	BLAST	HMM	Atchley	One-hot	Atchley + one-hot
Vip3 (108)	0	0	95	99	108
MACPF (5)	1	0	3	3	4
Toxin 10 (30)	0	0	10	17	21

﻿The column corresponding to each method shows how many proteins belonging to each category were picked by the corresponding method.

The table shows that our model managed to detect pore formers that were missed by traditional sequence homology approaches.﻿

We downloaded the sequences of the Vip3, MACPF, and Toxin 10 proteins from the Bacterial Pesticidal Protein Resource Center^31^. Our list of test proteins had 108 Vip3s, 5 MACPFs, and 30 Toxin 10 family proteins. For the tests we ran with the three protein families, we ensured that no homologs of the three families were present in our training set—that is, no Vip3s or Perforins or Toxin 10 s. To evaluate BLAST, we made a BLAST database out of our training set, and compared it with the test proteins. The E-value used was 0.01. The single hit for MACPF was due to the presence of thiol-activated cytolysins in the training set. To evaluate HMMs, we downloaded HMMs for each protein category in our training set from the PFAM database^32^, and evaluated if any of them could pick up proteins from our test list. The HMMs we downloaded included aerolysins, leukocidins, anemone_cytotox, colicin, endotoxin_c, endotoxin_h, hemolysin_n, and hlye (Hemolysin E). None of the HMMs we considered were able to pick up any of the proteins from the test categories—that is, HMMs are not geared towards picking up novel proteins. For our deep learning model, after training, the model was tested with the list of these proteins, and checked to see how many of these were picked up by the model as pore formers. As the table summarizes, our model managed to detect pore formers it was not trained on, even when traditional sequence homology-based approaches failed. Once again, the combined encoding method outperformed one-hot encoding and Atchley 5-factor encoding methods.

These results established that the model can short list pore formers it had not seen previously. We further tested the model with proteins of known function other than pore formation. We downloaded multiple enzyme classes from Brenda^33^ and ran it through our model. When we tested a list of approximately 1000 enzymes with Enzyme Commission (EC) numbers 1.9.1, 2.1.4, 3.8.1, 4.1.1, 5.1.1, 81% of them were classified as not being pore formers. For amidinotransferases with EC number 2.1.4.2, none of the 132 sequences were classified as a pore former. Table 2 lists samples of those proteins and their source and known annotation. None of these proteins was identified by the model as a pore former.

**Table 2:** Proteins with known functions other than pore formation that were correctly categorized by the model as not being pore formers.

Protein + annotation	Source
A0A518IH75|alcohol dehydrogenase (azurin)|EC 1.1.9.1|Planctomycetes bacterium Enr17|TrEMBL	Brenda^33^
Q8ZNC6|ornithine racemase|EC 5.1.1.12|Salmonella typhimurium (strain LT2/SGSC1412/ATCC 700720)|TrEMBL	Brenda
sp|P0DTQ2|DRDK_BACT7 5-deoxyribose kinase OS = *Bacillus thuringiensis* serovar kurstaki (strain ATCC 35866/NRRL B-4488/HD73) OX = 1279365 GN = drdK PE = 1 SV = 1	Uniprot^23^
sp|Q6HLF4|ILVC1_BACHK Ketol-acid reductoisomerase (NADP(+)) 1 OS = *Bacillus thuringiensis* subsp. konkukian (strain 97-27) OX = 281309 GN = ilvC1 PE = 3 SV = 1	Uniprot

## Conclusion

In this paper, we have outlined a computational approach for prioritizing novel pore forming proteins for further evaluation. The encoding method of choice, a combination of one-hot encoding and Atchley factors, gave us good accuracy ranges and receiver operating characteristic curves. It also allowed us to detect pore formers that were not part of the training set, unlike traditional sequence-homology approaches. Further improvements of the model will likely involve including additional protein features. However, keeping in mind the fact that the purpose of this model is to eventually evaluate hundreds of millions of proteins, a balance needs to be reached between the performance of the model and the feasibility of its implementation.

This approach will allow large numbers of protein sequences of unknown function to be evaluated for the possibility that they may have pore-forming properties. Ultimately this may lead to the discovery of entirely new families of pore-forming proteins, some of which may prove useful for controlling agricultural pests.

## Data Availability

The data and code outlined in this paper is available on request from T.W.K.

## References

[1] Mondal AK, Verma P, Lata K, Singh M, Chatterjee S, Chattopadhyay K (2020). Sequence diversity in the pore-forming motifs of the membrane-damaging protein toxins. J. Membr. Biol..

[2] de Maagd RA, Bravo A, Berry C, Crickmore N, Schnepf HE (2003). Structure, diversity, and evolution of protein toxins from spore-forming entomopathogenic bacteria. Annu. Rev. Genet..

[3] Palma L, Muñoz D, Berry C, Murillo J, Caballero P (2014). *Bacillus thuringiensis* toxins: An overview of their biocidal activity. Toxins.

[4] Chalivendra S (2021). Microbial toxins in insect and nematode pest biocontrol. Int. J. Mol. Sci..

[5] Jurat-Fuentes JL, Crickmore N (2017). Specificity determinants for cry insecticidal proteins: Insights from their mode of action. J. Invertebr. Pathol..

[6] Peterson B, Bezuidenhout CC, Van den Berg J (2017). An overview of mechanisms of cry toxin resistance in lepidopteran insects. J. Econ. Entomol..

[7] Tabashnik B, Brévault T, Carrière Y (2013). Insect resistance to Bt crops: Lessons from the first billion acres. Nat. Biotechnol..

[8] Storer NP, Thompson GD, Head GP (2012). Application of pyramided traits against Lepidoptera in insect resistance management for Bt crops. GM Crops Food.

[9] Doxey AC, Mansfield MJ, Montecucco C (2018). Discovery of novel bacterial toxins by genomics and computational biology. Toxicon.

[10] Wood V, Lock A, Harris MA, Rutherford K, Bähler J, Oliver SG (2019). Hidden in plain sight: What remains to be discovered in the eukaryotic proteome?. Open Biol..

[11] Torrieri R, de Oliveira FS, Oliveira G, Coimbra R (2012). Automatic assignment of prokaryotic genes to functional categories using literature profiling. PLoS ONE.

[12] Hanson A, Hanson A, Waller J, Crécy-Lagard V (2009). ‘Unknown’ proteins and ‘orphan’ enzymes: The missing half of the engineering parts list—And how to find it. Biochem. J..

[13] Altschul SF, Gish W, Miller W, Myers EW, Lipman DJ (1990). Basic local alignment search tool. J. Mol. Biol..

[14] Eddy SR (1998). Profile hidden Markov models. Bioinformatics.

[15] Kulmanov M, Khan MA, Hoehndorf R, Wren J (2018). DeepGO: Predicting protein functions from sequence and interactions using a deep ontology-aware classifier. Bioinformatics.

[16] Nauman M, Rehman H, Politano G, Benso A (2019). Beyond homology transfer: Deep learning for automated annotation of proteins. J. Grid Comput..

[17] Hou J, Adhikari B, Cheng J (2018). DeepSF: Deep convolutional neural network for mapping protein sequences to folds. Bioinformatics.

[18] Rifaioglu AS, Doğan T, Martin MJ, Cetin-Atalay R, Atalay V (2019). DEEPred: Automated protein function prediction with multi-task feed-forward deep neural networks. Nat. Sci. Rep..

[19] Alipanahi B, Delong A, Weirauch M, Frey BJ (2015). Predicting the sequence specificities of DNA- and RNA-binding proteins by deep learning. Nat. Biotechnol..

[20] Parker MW, Feil SC (2005). Pore-forming protein toxins: From structure to function. Prog. Biophys. Mol. Biol..

[21] Peraro MD, van der Goot FG (2016). Pore-forming toxins: Ancient, but never really out of fashion. Nat. Rev..

[22] Crickmore N, Berry C, Panneerselvam S, Mishra R, Connor T, Bonning B (2020). A structure-based nomenclature for *Bacillus thuringiensis* and other bacteria derived pesticidal proteins. J. Invertebr. Pathol..

[23] Uniprot. Available: https://www.uniprot.org/ (2020).

[24] Scikit-learn. Version 0.23.1. Available: https://scikit-learn.org/stable/.

[25] Python. Version 3.7.4. Available: https://www.python.org/.

[26] Keras. Version 2.3.1. Available: https://keras.io/.

[27] Sanahuja G, Banakar R, Twyman R, Capell T, Christou P (2011). *Bacillus thuringiensis*: A century of research, development and commercial applications. Plant Biotechnol. J..

[28] Wang G, Dunbrack JRL (2003). PISCES: A protein sequence culling server. Bioinformatics.

[29] Zhou, J. & Troyanskaya, O. Deep supervised and convolutional generative stochastic network for protein secondary structure prediction. In *Proceedings of the 31st International Conference on International Conference on Machine Learning* (2014).

[30] Atchley WR, Zhao J, Fernandes A, Druke T (2005). Solving the protein sequence metric problem. Proc. Natl. Acad. Sci..

[31] BPPRC. Available: https://www.bpprc.org/ (2020).

[32] Pfam database. Available: http://pfam.xfam.org/ (2020).

[33] Brenda: The Comprehensive Enzyme Information System. Available: https://www.brenda-enzymes.org/ (2020).

